# Phenolics: Occurrence and Immunochemical Detection in Environment and Food

**DOI:** 10.3390/molecules14010439

**Published:** 2009-01-19

**Authors:** Eline P. Meulenberg

**Affiliations:** ELTI Support VOF, Drieskensacker 12-10, 6546 MH Nijmegen, The Netherlands

**Keywords:** Phenolics, Environment, Food, Immunochemical detection, Isolation, Endocrine activity.

## Abstract

Phenolic compounds may be of natural or anthropogenic origin and be present in the environment as well as in food. They comprise a large and diverse group of compounds that may be either beneficial or harmful for consumers. In this review first a non-exhausting overview of interesting phenolics is given, in particular with regards to their presence in environment and food. For some of these compounds, beneficial, toxicological and/or optionally endocrine disrupting activities will be presented. Further, immunochemical detection and/or isolation methods developed will be discussed, including advantages and disadvantages thereof in comparison with conventional analytical methods such as HPLC, GC, MS. A short overview of new sensor-like methods will also be included for present and future application.

## Introduction

There is Dutch phrase ‘Meten is weten’, which means ‘To measure is to know’ and applies to the object of this review regarding the detection and optionally quantification of (poly)phenolic compounds using immunochemical methods. Phenolics comprise a very large group of both natural and anthropogenic compounds. Most natural phenolic compounds are secondary metabolites in plants and trees and as such are present in foods, but they are also used in additives, supplements and neutraceuticals.

With regard to their effects on organisms, industrial phenolics are inherently toxic, while natural compounds may show either negative or health-promoting activity. Many studies have been dedicated to assess the positive or negative effects of phenolics on organisms, including humans. In addition, efforts have been focussed on analytical methods for detecting and quantifying such compounds in the environment, in foods/feeds and in bodily fluids. In the following an overview is given of (poly)phenolic compounds, classified into industrial and natural; their potential toxicology, target levels and extraction methods used; beneficial and endocrinological effects; and analytical methods, including chemical, biological, immunochemical, binding assay and enzyme assays. Because this review is focussed on immunochemical methods, the principles of immunoassay are explained, its various formats are exemplified and advantages as well as disadvantages in respect to conventional analytical methods are given. Binding assays are discussed as a variant of bioassays / immunoassays because these may be used in the assessment of endocrinological effects of target compounds. In a subsequent section, various immunoassays reported in literature are discussed and our own experience with the production, evaluation and validation of antibodies, ELISAs, binding assays, immunosensors is described. Some recent developments are discussed regarding biosensors, in particular sensors based on antibodies, enzymes and/of binding proteins.

## Classification

The chemical group of (poly)phenolic compounds is, for the purpose of this review, classified into industrial and natural phenolics.

### Industrial phenolics

Phenol consists of a hydroxyl group attached to an aromatic C6-hydrocarbon ring. Phenol and its numerous derivatives and polymers are used extensively in industry in the fields of thermoset plastics, phenolic resins, polymer chemistry, wood industry, aerospace, building industry, automotive industry, abrasives, plasticizers, cleaning products, pesticide manufacturing, detergent applications, etc. [1,2]. It will be appreciated that for the purpose of this review only those compounds that may enter into the environment in free form are of interest. This means that larger end products are not included (extensive recycling), but merely small final compounds, intermediates, degradation products, excretion products and the like that may be found in the environment, food and/or organisms. Some examples of industrial phenolics comprise alkylphenols, nitrophenols, alkoxyphenols, halogenated phenols, nonoxynol-9, nonylphenoxyacetic acid, butylated hydroxytoluene, alkylethoxylates, bisphenolic compounds, Triton^®^ X-100, *p*-cresol, pyrocatechol, methylcatechol, chlorophenoxy acids, etc. Examples of industrial and agricultural phenolics found in the environment, food and organisms that have been the subject of extensive research are listed in Table 1, including some specific examples of special interest.

**Table 1:** Industrial phenolics.

Alkylphenolethoxylates	Alkylphenols
Bisphenolics	Bromophenols
Butylated hydroxy toluene	Chlorophenols
Chlorophenoxy acetic acids	Dialkylphenols
Fluorophenols	Hydroxylated PCBs
Hydroxyanisole	Hydroxytoluene
Methoxyphenols	Methylcatechol
Nitrophenols	Nonoxynol-9
Nonylphenol	Nonylphenoxy acetic acid
Octylphenol	p-Cresol
Pentachlorophenol (PCP)	Pyrocatechol
Trichlorophenol	Triton^®^ X-100

### Natural phenolics

As mentioned above, natural phenolics are secondary plant substances, i.e. not central to metabolism. Their functions are not always known, but some are colouring agents and others are potentially protective, not only for the organism of origin but also as isolates in medicinal products, supplements and/or neutraceuticals. Chinese physicians have been using plant phenolics for ages to treat various diseases and disorders. Nowadays more than 8,000 phytochemicals are known, of which more than 5,000 are flavonoids. They are divided into at least 10 types, depending on their basic structure: phenols, phenolic acids, hydroxycinnamic acids, coumarins/isocoumarins, naphthoquinones, xanthones, stilbenes, anthraquinones, flavonoids and lignins [3]. Some examples are listed in Table 2. Included are also some natural phenolic compounds derived from fungi such as *Penicillium, Aspergillus* and *Fusarium*. These organisms may grow on crops like cereals and, under certain circumstances release very toxic compounds, mycotoxins.

**Table 2:** Natural phenolics (phytochemicals).

4-Hydroxycinnamic acid	4-Hydroxycinnamoyl quinic acid	Acacetin
Baicalein	Baicalin	Brassica sterol
Caffeic acid	Campesterol	Canabinoids
Cinnamic acid	Citrinin	Coumaric acid
Coumestrol	Cyanidin	Daidzein
Delphinidin	Dihydroxybenzoic acid	Divanillyltetrahydrofuran
Dopac	Ellagic acid	Enterodiol
Enterolactone	Epicatechin	Equol
Ergosterol	Esculin	Ferulic acid
Formononetin	Fraxetin	Gallic acid
Gentisic acid	Guaiacol	Gentisic acid
Hesperetin	Hydroxybenzoic acid	Hydroxytyrosol
Kaempferol	Kurarinone	Lignin
Malvidin	Matairesinol	Morin
Mycophenolic acid	Myrecitin	Nivalenol
Nordihydroguaiaretic acid	Ochratoxin	Pelargonidin
Peonidin	Petunidin	Phenol carbonic acids
Phlorotannins	Protocatechuic acid	Prunetin
Psilocin	Puerarin	Quercitin
Resveratrol	Rutin	Salicylic acid
Secoisolariciresinol	Sinapinic acid	Sissotrin
Sitosterol	Stigmasterol	Tannins
Taxifolin	Taxol	Thymol
Todolactol	Tyrosol	Vanillic acid
Zearalenone	Zeranol	

### Endogenous and pharmaceutical phenolics

In the context of this review, natural phenolics include endogenous and pharmaceutical steroids such as estradiol, ethynyl estradiol, DES (diethylstilbestrol), thyroid such as T4 and T3, paracetamol, certain antibiotics, vitamins, etc., because these are also detected in the aqueous environment or food [4,5,6,7,8,9]. Similarly, certain endogenous neurotransmitters are phenolics that may excreted in the urine and thus enter into the environment.

**Table 3:** Endogenous and pharmaceutical phenolics.

Beta-agonists	Catecholamine
Collinomycin	Epinephrine
Estradiol	Estriol
Diethylstilbestrol	Dopamine
Ethynylestradiol	Frenolicin
Methoxytyramine	Mycophenolic acid
Paracetamol	Parvaquone
Pasiniazolide	Pratensin
Prenaterol	Tocopherols
Tocotrienols	

## Effects of phenolics

### Toxic effects

Industrial phenolics inherently are toxic and some are bioaccumulated in the food chain. Toxicity assessment includes whole organisms, tissues, cells, receptors, DNA and enzymes. Hereinafter analytical methods are described as well as concentrations found.

### Beneficial effects

Health-promoting effects of phytochemicals have been studied for centuries in Chinese medicine and nowadays especially with regard to antioxidant, anticancer and anti-angiogenesis agents, as means for reducing menopausal symptoms in women, for reduction of LDL and as a consequence cardiovascular diseases, as anti-osteoporotic, anti-angiogenesis or anti-tumour agents and many others. Most of these effects can be converted to hormonal/endocrinological effects, as explained in more detail below.

### Endocrinologic effects

Some of the industrial and natural phenolics exhibit effects on hormonal systems in animals, including humans. For that reason they are called endocrine disruptive compounds (EDC). EDC may act as estrogenic, androgenic, progestagenic, thyroidal, glucorticoidal agonists or antagonists. In particular those compounds that show estrogenic action have been subject of many recent studies. This particular class of phytochemicals is referred to as phytoestrogens, of which the majority belongs to the group of flavonoids. Members of the group of flavonoids, showing the most potent estrogenic activity, fall into three chemical classes: coumestans, prenylated flavonoids and isoflavones, and non-flavonoid phytoestrogens comprise the lignans (COT Working Group on Phytoestrogens). Phytoestrogens have been shown to possess weak affinity for the estrogen receptor, however due to their relatively high concentrations in certain foods and as a consequence in blood, their estrogenic effects may be comparable to endogenous estrogens. In some cases, however, they may even act anti-estrogenic. In this regard they are interesting in estrogen/androgen-related diseases and cancers. In recent years also the thyroidal and glucocorticoidal effects of environmental pollutants and phytochemicals have gained interest, mainly because the presence of pollutants in humans might explain the increase in modern diseases and disorders, especially in Western countries, whereas the intake of phytochemicals may prevent these diseases. Interference with endogenous hormone systems may be related with the increase of the incidence of metabolic syndrome, obesity, eating disorders, diabetes, cardiovascular diseases, Alzheimer, depression, memory deficits, developmental disorders, bone disorders, etc. The relation between the glucocorticoid hormone cortisol and fat deposition is most obvious in the disorders Cushing syndrome and Addison’s disease [10]. In the first, cortisol concentrations are very high and related to increased body fat; in the latter, cortisol concentrations are extremely low and related to decreased body fat. There are also strong indications of the relation between glucocorticoids and diabetes or insulin resistance.

## Hormonal systems in humans

**Figure 1:**
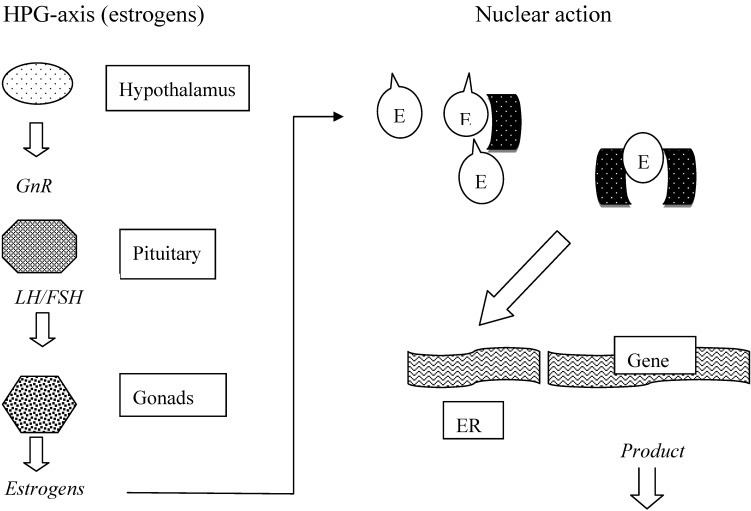
Hypothalamus-pituitary-gonadal pathway.

In the human body, one of the hormonal pathways comprises the hypothalamus-pituitary-gonadal axis (HPGA). Hereby sexual development and functioning such as reproduction is regulated involving several protein and steroid hormones in a negative feedback system. The main active compounds are estradiol and testosterone being produced in the gonads, transported in the circulation to their target organs, taken up by cells, bound to their respective receptors and moved to the nucleus in dimerized form where they bind to the so called responsive elements leading to the synthesis of proteins and regulating factors. A schematic overview of the HPAA is given in Figure 1.

Due to their chemical similarity with estradiol, several phyoestrogens may mimic or counteract the action of estradiol. Thereby they may have an influence on various levels in the HPGA: i) in the feed-back regulation, they may inhibit the synthesis of steroids, e.g. by an effect on hydroxylases, dehydrogenases, aromatases; ii) at the level of transport they may have an affinity for the transport protein SHBG, Sex Hormone Binding Globulin, where they may displace the endogenous steroids and increase the free, biological active fraction; iii) at the level of intracellular receptors they may act agonistic or antagonistic in relation to the binding of the endogenous steroid; iv) at the level of degradation they may influence the enzymes involved in catabolism or the synthesis of proteins in the liver. Many studies have been focussed on the effect of phytoestrogens on cancer cell lines and/or the binding to the estrogen receptor (ER) in the scope of hormone-related cancers such as breast and prostate cancer.

In addition to the HPGA, the HPAA (Hypothalamus Pituitary Adrenal Axis) represents an important feedback mechanism regulating (gluco)corticoid and mineralocorticoid hormone synthesis and actions. The main glucocorticoid hormone is cortisol, also referred to as the stress hormone because its levels dramatically increase in stress situations. Moreover, cortisol is implicated in psychological and mental disorders like depression; further cortisol is implicated in metabolic disorders such as diabetes, an increasing health problem in western countries. Corticoids are produced in the adrenals glands, released into the circulation, bound by CBG (corticosteroid binding globulin, the main transport protein in blood), transported to target cells, internalised and bound to the glucocorticoid receptor, taken up by the nucleus, bound to responsive elements and finally leading to the synthesis of mRNA, proteins and other factors, the whole being regulated in a feedback fashion. Although mineralocorticoids, of which aldosterone is the main hormone, are part of this system as being produced by adrenals, their target organs and regulation is different, but there is a strong interaction with glucocorticoids, in particular with regard to the renal water and salt balance and related disorders due to a comparable affinity of aldosterone and cortisol for the mineralocorticoid receptor (MR).

The last hormonal system comprises the HPThA, the Hypothalamus Pituitary Thyroid Axis. In this feedback system the thyroid hormones T4 (thyroxine) and T3 (triiodothyronine) are the principle hormone components. In this case T4 and T3 are produced by the thyroid glands, secreted and transported by TBG (thyroid binding globulin) and TTR (transthyretin), intracellularly bound to receptors, to response elements in the nucleus and processed in the translation and transcription machinery. Thyroid hormones are highly implicated in development, metabolism, mental conditions such as learning and possibly ADHD, etc. Various environmental pollutants have been shown to possess thyroidal activity and have been subject to various studies [11,12,13,14,15].

In many textbooks, the hormonal systems have been extensively described, in particular with regard to human beings and clinical diagnostics. Regulation and actions of both steroid and thyroid axes are similar to the one presented in Figure 1. It should also be emphasized that there exist strong interrelations between the different hormonal systems, which makes it sometimes difficult to assign a particular effect of an exogeneous substance in a subject.

## Analytical methods

Various methods have been developed for the detection and quantification of (poly)phenolic compounds, including HPLC, GC, LC-MS, GC-MS, TLC, CE, paper chromatography. These are the conventional methods used in analytical chemistry. Because such methods are beyond the scope of this review, the reader is referred to some excellent reports [16,17,18,19,20,21,22]. For example, Guardiola *et al.* have reported about the GC and HPLC separation of cholesterol and phytosterol oxidation products [16]. Methods and assay conditions for the determination of anthocyanins in plants using various techniques have been reviewed by Mazza *et al.* [17]. Two reviews of the application of capillary electromigration methods for food and environmental analysis, including phenols, polyphenols, pesticides and antibiotics, were given by Cifuentes [18] and Dabek-Zlotorzynska and Celo [19]. The determination of polyphenols by electromigration methods was the subject of a review by Jáč *et al.* [20]. The separation and identification of lignans and flavonoids using chromatographic methods such as GC, HPLC and TLC was reviewed by Willför *et al.* and de Rijke *et al.* [21,22]. A further report about the application of LC-MS/MS for the measurement of phytoestrogens, including isoflavones, metabolites, lignanes and flavanones, in human urine and serum was published by Grace *et al.* [23]. General methods and applications of conventional analytical methods for the detection of phenolic acids and flavonoids in natural products were reviewed by Stalikas [24]. The strengths of these conventional methods, especially GC, HPLC and MS, are the measurement many compounds simultaneously, at high sensitivity, and the possibility to identify unknown compounds. Their weakness is the laborious clean-up required, expensive equipment and need for trained technicians.

## Biological methods

In addition to conventional analytical methods, biological methods have been developed for the assessment of effects at the cellular, enzymatic or receptor level, as well as for the determination of concentrations of industrial phenolics and phytochemicals as well as metabolites thereof in the environment, food or biological fluids. These include immunoassays, binding assays and biosensors as discussed in the following.

### Cell culture

Because of the concern for human health effects, various cell culture systems have been used to assess toxicity of phenolics as for example disruptors of cell membrane integrity, cell metabolism, division and proliferation, intracellular signalling. Cell culture systems include normal, tumour and engineered cells such as hepatic cells, breast cancer cells, bone cells, fibroblasts, skin cells and several other cell lines of which many are available from the American Type Culture Collection (ATCC) orATCC-LGC standards partnerships, Universities or specialised companies. One of the techniques for the assessment of toxicity is QSAR (quantitative structure-activity relationship). According to the results of Wright and Shadnia [25], there is a strong relation between log P, pK_a_, OH bond dissociation enthalphy (QSAR descriptors) and cytotoxicity of substituted phenols. It should also be noted that some metabolites of phenolic compounds are more toxic than their parent compounds because most metabolites are hydroxylated and thus more soluble and bioavailable. In addition, the well-known cytotoxicity, mutagenicity, genotoxicity tests may be used to assess toxicity such as the Ames test (gene mutations), SOS, UmuC, Mutatox and Comet (DNA damage), Microtox (luminescent marine bacteria), Calux, and Vitotox (genotoxicity) [26,27,28,29,30,31,32,33,34,35]. 

### Receptor assays

Different from direct cell toxicity, many compounds exert their effect through intracellular receptors. The number of intracellular receptors is limited and in the scope of this overview mainly the steroid/thyroid receptors are discussed. Binding of endogenous steroid/thyroid hormones is always followed by dimerisation of the particular receptor, whereupon several factors are recruited and the complex is transported into the nucleus. Here it is bound to responsive elements, translated into mRNA and transcribed into proteins or regulation factors. Endogenous and non-endogenous compounds of interest may either have an agonistic or antagonistic effect in this pathway. To gain insight into putative effects of phenolics, receptors may be isolated and used in a receptor binding assay to assess affinity. Such assays have been applied in clinical, pharmacological and environmental research. Nowadays various receptors and receptor kits are commercially available and have been described in literature. Because affinity of a certain compound for a particular receptor does not give a decisive answer about any agonistic of antagonistic effect, receptor genes and responsive elements coupled to reporter genes have been manipulated into both prokaryotic and eukaryotic cells. Such cell lines are used for effect and dose-response studies. Some examples of commercially available kits are the E-Screen, YES assay, Rikilt yeast assay, Calux (BDS) assay, etc. [36,37,38,39,40,41,42]. The incorporated reporter genes lead to a detectable signal when an added compound has an effect on the receptor gene. The relative hormonal effect can be calculated from the amount of signal of the compounds tested. An overview of receptor-based assays suitable for food and environmental EDC analysis was given by Holland (2003) [37]. Mammary cell lines incorporated with genes responding to glucocorticoids, progestagens, androgens or estrogens were used by Willemsen *et al.* for EDC analysis. Several endogenous, pharmaceutical and drugs of abuse were tested, wherein the EC50 was sometimes very low indicating high affinity [38]. The phytoestrogen genistein was detected in river waters using a ER-containing recombinant yeast assay by Kawanishi *et al.* [39]. Samples contained from 0 to 72 pmol/L (0-19.6 ng/L) of genistein, expressed in E2-equivalents. A similar yeast estrogen bioassay was developed at the Rikilt Institute in Wageningen (The Netherlands) and validated for feed and food [40]. In addition to cell lines incorporating manipulated receptors, isolated receptors were also used in competitive assay format in order to determine the affinity of target compounds. Thus both the androgen and estrogen receptor in conjunction with the corresponding tritiated ligand were used in competitive assays for the assessment of the affinity of several contaminants [41]. Of the compounds tested, PCP and nonylphenol, for example, bound to the androgen receptor. The so-called ELRA, (Enzyme Linked Receptor Assay) for estrogenic and androgenic compounds was developed in the scope of bioresponse-linked analyses [42]. This assay is based on the hER-alpha or the human androgen receptor that were isolated from appropriate cell cultures and they showed a detection limit of below 0.1 µg/l for estradiol and below 1 µg/l for testosterone. Displacement curves allowed the determination of effects of spiked tap ware, river water and sewage effluent. Unknown compounds can be identified by coupling affinity extraction step and detection by LC-MS-MS.

With regard to receptor analyses, Scippo *et al.* [43] performed an investigation into the affinity of a large series of chemicals and phytoestrogens for the human estrogen, progestagen and androgen receptor using radioactive tracers in a competitive format. Several insecticides, herbicides, industrial chemicals and phtoestrogens were tested for binding capacity. In particular, nonylphenol, bisphenol A (BPA), some isoflavones and flavonoids were found to bind to hER-alfa. An overview of receptors in relation to *in vivo* toxic effects of contaminants can be found in Janošek *et al.* [44]. 

Further receptor studies include the retinoid X receptor as well as the farnesoid, glucocorticoid and estrogen receptor that may involved in epidemics of diseases related to metabolic dysfunction [45,46]. Several pollutants have been shown to have an effect through these receptors by perturbing corresponding signalling pathways, such as DES, BPA, phthalates and organotins. Such compounds are called obesogens, because they affect e.g. adipocyte proliferation and adipogenesis.

### Enzyme assays

In any organism pollutant phenolic compounds may also have an effect on one or more enzymes, both stimulative or inhibitive. Because enzymes play importants roles in various hormonal pathways, isolated enzymes have been used to determine effects at this level. For example, the enzymes of the cytochrome P450 family are involved in liver metabolism and steroid hormone pathways. Similarly, several dehydrogenases affect steroid pathways, including 3ßHSD, 11ßHSD, 17ß-HSD, 21-HSD. With regard to glucocorticoids and mineralocorticoids the enzymes 11ß-HSD 1 and 2 are important components in regulation. These enzymes form an oxidase-reductase system that interconverts cortisol and cortisone, of which cortisol is the active and cortisone the inactive glucocorticoid hormone. In kidneys, water and salt homeostasis is regulated by aldosterone and mineralocorticoid receptors. Besides aldosterone also cortisol binds to the receptor thereby displacing aldosterone and inhibiting its function. The enzyme 11ß-HSD2 is present in kidney tissue and converts cortisol into cortisone. With regard to environmentally relevant chemicals and phytochemicals, several have been shown to inhibit 11ß-HSD2 and can thus lead to hypertension, hypokalemia and heart failure [47,48]. Similarly, the same chemicals were tested for inhibition of MR translocation or 11ßHSD1 activity. Some of these chemicals are worth mentioning: abietic acid, fusidic acid, gossypol, magnolol, tea polyphenols, zearalenone, octylphenol, nonylphenol, BPA and some phthalate derivatives. Disruption of 11ßHSD activity has been implicated in the HPAA regulation and various diseases and disorder.

A major health problem in this century is obesity. In this disease several hormonal factors play a role, among which corticosteroids. Enzymes involved in glucocorticoid synthesis and conversion of cortisol into cortisone and vice versa, have therefore been subject of studies concerning the influence of various xenobiotic compounds. Thus it was found that the isoflavones genistein and daidzein inhibited 21-hydroxylase and 3ß-hydroxylase resulting in reduced cortisol synthesis. In addition, flavanone and some monohydroxylated flavone derivatives selectively inhibit 11ß-HSD1; glycyrrhizine, a sweetener and casing material, inhibits 11ß-HSD2. Due to these actions such compounds either decrease or increase cortisol concentrations and thereby exert a positive or negative effect, respectively, on the onset of diabetes [49]. Cortisol production also is inhibited by PCB and DDT metabolites through 11ß-HSD [47].

With regard to thyroidal effects an anti-thyroid effect of dietary flavonoids an isoflavones has been described by Divi and Doerge and Divi *et al*. [50,51]. These compounds, in particular genistein and daidzein, inhibit thyroid peroxidase that is involved in thyroid hormone synthesis.

Enzyme assays to assess activity of compounds of interest are generally performed in solution using the particular enzyme in isolated form. Tyrosine kinases, aromatases, hydroxylases, steroid converting enzymes and acetylcholine esterases may be used to investigate the influence of both anthropogenic and natural phenolics on steroid and thyroid pathways.

### Immunoassays

**Figure 2:**
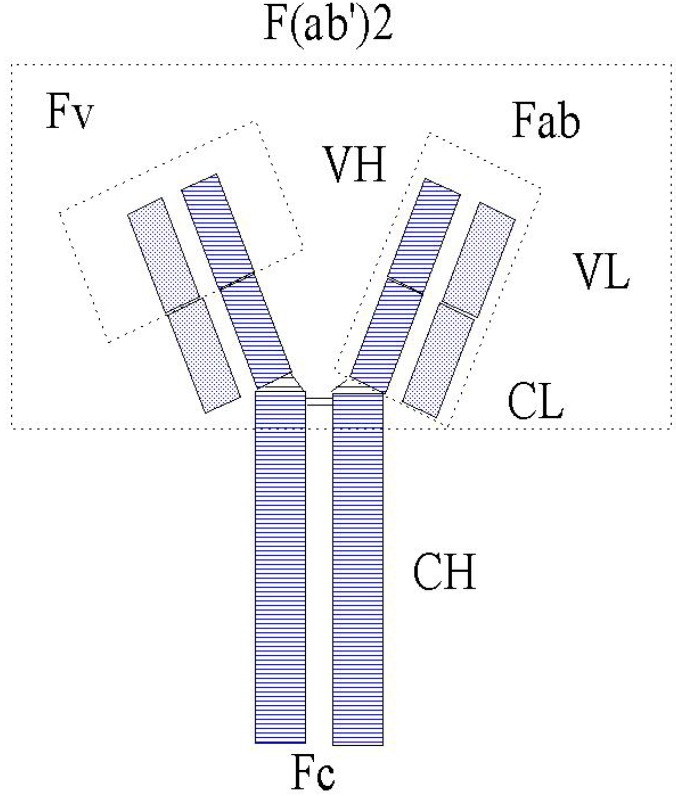
Schematic representation of an antibody (IgG).

Legend: Fc = constant fragment, Fv = variable fragment, CH = constant heavy chain, CL = constant light chain, VH = variable heavy chain, VL = variable light chain, Fab = antigen binding fragment.

For the purpose of this review wherein immunochemical methods are the central subject, the principles of immunoassay are described in more detail. Although some people consider the immunoassay as biological because it is based on a protein, in fact it is a reversible chemical reaction between an antibody and an antigen forming a complex. The antibody is the main component in this reaction and originates from the defence system of an organism. As soon as a foreign substance, the antigen, enters the body, the defence system is triggered to produce antibodies by the B-cells to be secreted into the circulation. The mechanism of antibody production comprises at first the rearrangement of genes that code for the light and heavy chains of an antibody which are finally combined to a whole antibody protein linked by disulfide bonds (schematically shown in Figure 2). Further events that may affect antibody generation and diversity are found in literature and art-related text books.

The number of genes available in the genome and the numerous different rearrangements provides for the potential of the enormous diversity of antibodies that each are specific for one antigen. The constant fragment (Fc) of the antibody renders species specificity to the protein, whereas the variable fragment (Fv) forms the cavity where the antigen is bound with high affinity in order to be eliminated from the body. In the 1970s researchers recognized the potential of using antibodies for the determination of antigens in blood and the immunoassay was born. After isolating antibodies (Ab), the first immunoassays were performed in a reaction tube using labelled signalling antigen (Ag*) for the detection of the formed immune complex as shown in the formula below:

Ab + Ag + Ag* ⇔ Ab-Ag + Ab-Ag*

For quantification, the bound and free fraction of the reaction mixture is separated and the bound fraction counted, where higher antigen concentrations are inversely proportionally related to the amount of bound tracer (labelled antigen).

Initially, antibodies were experimentally produced in laboratory animals such as rat, rabbit, sheep, horse, chicken etc, by injecting them with target antigen and after some weeks isolating the desired antibodies (IgG) for use in immunoassays. Such antibodies are referred to as polyclonal because an antiserum contains several different populations of antibodies each with its own characteristics with regard to specificity and affinity. In 1975, Köhler and Milstein developed the hybridoma technique for the production of monoclonal antibodies [52]. In this case, antibody-producing cells from immunized mice are fused with myeloma cells yielding hybridoma cells that have combined the characteristics of both, i.e. antibody synthesis and infinite division. After the fusion the resulting cells are diluted to single cells and cultured. Thus each cell culture may produce one single type of antibody in unlimited amounts.

**Figure 3:**
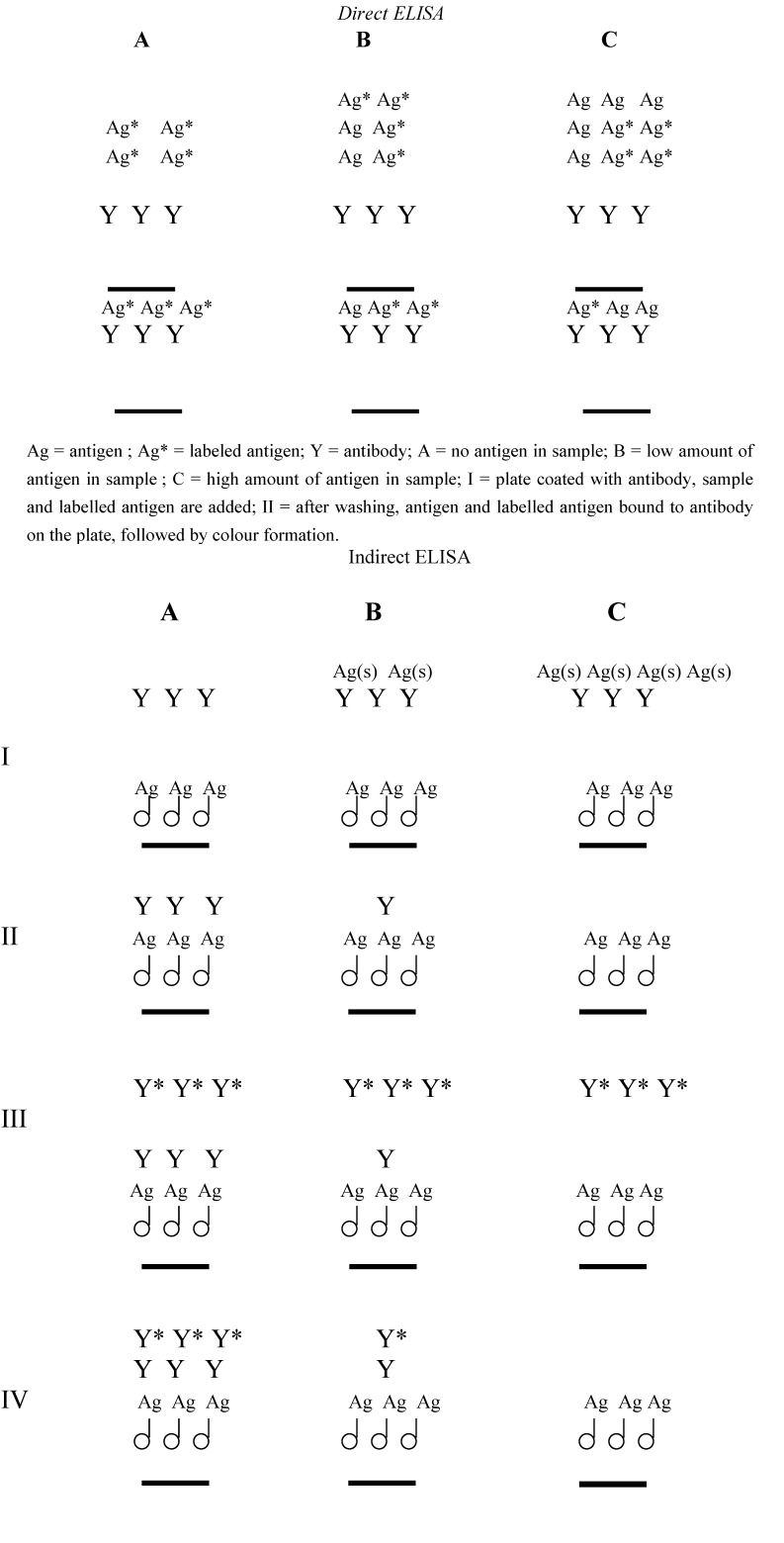
Principle of the direct and indirect immunoassay.

Immunoassays have been extensively used in clinical diagnosis due to the potential to detect target compounds such as hormones in very low concentration in complex sample matrices. At the end of the last century this property was adopted for environmental analysis and has led to an increasing number of publications at this field. In the course of time, various different formats for immunoassay have been developed including direct and indirect assay; heterogeneous and homogeneous assay; radioactive, enzymatic, fluorescent, chemiluminescent assays, solution-based and solid support-based assay; and recently immune-biosensor assays. The format used most frequently is the so called ELISA, Enzyme Linked Immuno Sorbent Assay. Because of its importance, a schematic representation of both a direct and an indirect ELISA is given in Figure 3.

Ag = Antigen bound to carrier protein coated onto the plate; Ag(s) = antigen in sample; Y = antibody; Y* = labelled second antibody; A = high amount of antigen in sample; B = low amount of antigen in sample ; C = no antigen in sample; I = plate coated with antigen bound to carrier protein, antibody and sample are added; II = antibody bound to the plate after the immunochemical competition reaction and washing; III = labelled second antibody is added; IV = labelled second antibody bound to the plate, followed by colour formation.

In the direct ELISA the tracer is antigen covalently coupled to an enzyme such as a peroxidase. Antibody is coated onto the wells of a microtiter plate, then standard/sample and tracer are added for incubation. After completion of the immune reaction the supernatant containing the free, unbound fraction is discarded and to the bound fraction in the wells enzyme substrate and chromogen (change in colour by the enzymatic reaction) is added. Colour formation is measured by spectrophotometric analysis. Then, the sample concentrations of the target antigen can be calculated using the standard curve plotted. In the indirect ELISA the antigen is coated on the wells of a microtiter plate, then sample and antibody are added for incubation, i.e. competition of bound and sample antigen for binding sites on the antibody. After the reaction the supernatant is discarded and the bound fraction is quantified. There are several ways for quantification in the indirect ELISA. Either the antibody labelled with enzyme is used in the reaction, or a labelled second antibody raised against the primary antibody is used for colour formation and measurement.

Immunoassays have been applied at many fields and for numerous target compounds ranging from small hormones or environmental pollutants to microorganisms and eukaryotic cells, e.g. in immunohistochemistry and cancer therapy. Advantages of immunoassay are its speed (generally an ELISA may be completed in two hours), specificity, sensitivity (in the nanomolar and sometimes femtomolar range), the possibility to measure many samples at the same time, its low costs, in particular with respect to conventional analytical methods such as HPLC, GC and MS, and the ability for automation and high-throughput measurements. A drawback of immunoassay may be again its specificity, because one antibody is only specific for and can be used for the measurement of one antigen or a group of related antigens, in contrast to conventional analysis. Another point of interest is the size of the antigen. In order to be able to elicit an immune reaction in the animal, the target compound should be > 500 Daltons and show a 3D configuration (linear molecules generally do not elicit an immunological reaction). Compounds being recognized by antibodies, but too small to raise an immunological antibody reaction, are called haptens. That means that small compounds such as lower alkylphenol, pesticides, vitamins, etc. will not be able to trigger the synthesis of antibodies. To solve this problem in immunisation, the particular hapten is covalently coupled to a carrier protein before immunisation. Carrier proteins typically used comprise BSA (bovine serum albumin), KLH (Keyhole Limpet Hemocyanin) and/or thyroglobulin. Conjugation of carrier proteins to antigens/haptens requires functional reactive groups on the hapten or in case these are missing, the derivatisation of the parent compounds to include such functional groups without changing the physico-chemical properties as target compounds too much. The chemistry of coupling and derivatisation as well as the use of spacer moieties between hapten and carrier protein has been extensively described in literature, textbooks and manuals [53,54,55,56]. In addition, various coupling kits are commercially available. The same technique is used for the generation of enzymatic, fluorescent, chemiluminescent tracers. It will be appreciated that in the indirect immunoassay where antigen/hapten is coated onto the wells of a microtiter plate, the same difficulty is encountered. In this case the antigen/hapten is similarly conjugated to a carrier protein, typically OVA (ovalbumine) or BSA, to allow for coating onto a solid phase, such as the wells of a microtiter plate.

The dimension of the antigen-binding cavity of an antibody is suited to accommodate compounds of up to 6 nm in diameter. This means that small haptens may be completely surrounded by the antigen-binding site of the protein, whereas larger peptides and proteins fit in only partly. The part of a larger compound that is recognised by the antibody is called epitope. It will be recognized that large molecules such as (poly)peptides and proteins may possess many epitopes and hence lead to broad populations of antibodies during immunisation. In contrast, antibodies raised to haptens can be highly specific for one particular compound, although members of the same chemical class are sometimes also recognized and bound with more or less the same affinity. This means that cross-reactions present an important factor in the development of immunoassays for haptens. The principles of immunoassay, development, hapten and conjugate design and validation have been extensively described in literature and patents. Before using an antibody or immunoassay, it should be validated by determining several parameters according to e.g. ISO norm 15089. These parameters comprise detection limit, working range, precision, accuracy, recovery, cross-reactivity/specificity, matrix effects. Antibodies may be either compound-specific or group-specific which defines their uses. For example, in diagnostic application a compound-specific assay is highly desired, whereas for screening purposes a group-specific assay may be more suitable as in early warning for water quality control.

### Application of immunoassays

**Figure 4:**
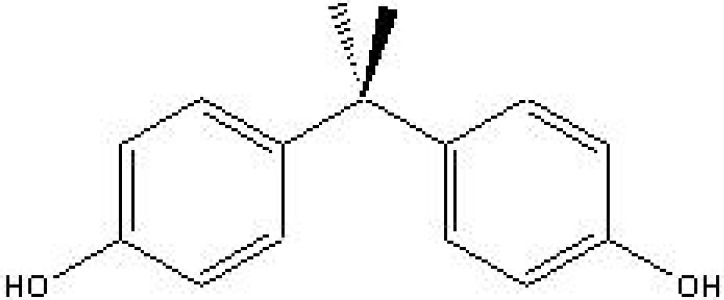
Chemical structure of bisphenol A.

In view of the effects of natural and synthetic phenols as well as their presence in the environment, food and biological fluids, immunoassays are suitable for detection, quantification, early warning and monitoring purposes. For example, in the scope of water quality control of surface and ground water used for the production of drinking water various immunoassays have been developed, including those for the detection and quantification of industrial pollutants such as alkylphenols, bisphenol A, pesticides such as chlorophenoxy acetic acids, and many others. To illustrate the development and validation of environmental immunoassays, two assays are described: a highly specific ELISA for bisphenol A (BPA) (Figure 4) and a group-specific ELISA for nonylphenol (NP)/octylphenol (OP).

Molecular weight of BPA is 228 g/mol and for that reason the derivative bisphenol valeric acid (BVA) was used for the synthesis of the immunogen by coupling it to BSA. Similarly, BVA was conjugated to horseradish peroxidase (HRP) for use as an enzymatic tracer and to OVA for coating in the indirect format. Polyclonal antibodies were produced in rabbits and used in a competitive ELISA [55]. The parameters determined in the validation of the ELISA are presented in Table 4.

**Table 4:** Parameters of one ELISA for BPA.

Immunogen	Bisphenol Valeric Acid-BSA
Tracer	Bisphenol Valeric Acid-HRP
Detection limit	0.035 nM
Working range	0.1 – 1000 nM
Precision	
Intra-assay	12.3 % C.V.
Recovery	79 – 121 %
Cross-reactivity (%)	*
Bisphenol A	100%
4,4'-(ethylidene) bisphenol	10%
Bis-(4-hydroxy phenyl)-methane	1%
Nonylphenol	1%
4-cumylphenol	20%
Vinclozolin	0.1%
Pirimifos-ethyl	< 0.1%
17ß-Estradiol	< 0.1%
2,4-D	0.1%
Sulfadimidine	< 0.1%

* = depending on the matrix.

From the above it can be concluded that this BPA ELISA is very sensitive and highly specific. Therefore, it can be used in screening of surface water to assess its presence and levels for water quality control. BPA is an important pollutant in that it is used in high amount in industry and released into the environment. Further, due to its use as a plasticizer it diffuses from plastic and coating materials and thus it may pollute foods and can be ingested as such. It has been shown to possess estrogenic activity and in particular poses a risk for newborns when it is present in baby food. [58,59,60,61,62]. 

The alkylphenols nonylphenol and octylphenol are predominantly derived from ethoxylates used as detergents. These compounds too have been shown to possess estrogenic activity [59,63,64]. For the development of an ELISA, two derivatives were used for coupling to BSA and HRP: COOH-C8-alkylphenol and COOH-C6-alkylphenol. Polyclonal antibodies were raised in rabbits. The assay was validated as above and the results for one of these are presented in Table 5. From the cross-reactivities measured it is obvious that this antibody is group-selective and may be used for general screening of samples for the presence of alkylphenols [57].

Sewage sludge has long been used a fertilizer for soils and farmlands, but this application has been banned due to the presence of contaminants, among which alkylphenols. The presence of alkylphenols in soil as a consequence of such use has been determined and ingestion by grazing ruminants as well as tissue or milk concentrations in sheep has been described [65,66,67]. 

Trichlorophenol is listed as priority pollutant by the US Environmental Protection Agency (EPA) and the EU due to its toxicity, carcinogenicity, persistence and bioaccumulation. It is used in insecticidal, bactericidal and antiseptic formulation and as an intermediate in various production processes. A competitive immunoassays has been developed by Galve *et al*. [68] showing a detection limit of 0.2 µg/L and good accuracy and it can be used to analyze trichlorophenol in drinking water. Adaptation of this immunoassay for urine measurements allowed for the potential assessment of a relation between urinary chlorophenols and occupational exposure [69].

**Table 5:** Parameters of ELISA for AP.

Immunogen	BSA-C6-alkylphenol
Tracer	HRP-C6-alkylphenol
Detection limit	1 nM
Working range	2 – 100 µM
Precision	
Intra-assay	1 – 50 % C.V.
Recovery	20 – 95 % *
Cross-reactivity (%)	
Octylfenol	100%
4-tert-Butylphenol	6%
2-sec-Butylphenol	1%
4-pentylphenol	556%
4-n-heptylphenol	256%
4-n-propylphenol	33%
2-n-propylphenol	<0.1%
4-Isopropylphenol	6%
4-n-hexylphenol	300%
4-chloro-2-cyclo-hexylphenol	< 0.1%
Nonylphenol (tech)	12%
4-n-nonylphenol	30%
Bisphenol A	8%

* = depending on the matrix.

Pentachlorophenol (PCP) is a compound used as preservative for wood and as pesticide in agriculture. Immunoassays have been developed for screening application at waste sites, in surface, drinking and groundwater and in soils. [70,71]. The sensitivity of these assays varied from 0.125 mg/l with a polyclonal antibody to 30 µg/l with a monoclonal antibody. In both cases there was some cross-reactivity with trichloro- and tetrachlorophenols. Results were compared to GC EPA 8040/604 and GC-MS EPA 3540/8270 methods. An immunoassay kit was subsequently used for the rapid detection of PCP in water, soil and effluents and ELISA results were compared to LC-MS and LSE-MS. It appeared that pulp effluents contained up to 1.8 µg/L of PCP.

Mycophenolic acid (MPA) is a fungal antibiotic and due to its additional antiviral, antiparasitic and antitumor properties it has been used in medical applications. The MPA producer *P. Roqueforti* is found in food and feed and suspected in milk and cheese. An ELISA for detection has been described by Usleber *et al.* [72] showing a detection level of 100 pg/mL in milk and 0.5 ng/g in cheese, and in several types of cheese concentrations of up to 1200 ng/g were detected. Of course many more immunoassays have been developed for environmental analysis of anthropogenic pollutants. Those that are relevant for this review are summarized in Table 6.

**Table 6:** Additional immunoassays for industrial pollutants.

Target Compound	Format	Application	Reference
Trichlorophenol	ELISA	ELISA	[73]
Nonylphenol	ELISA	Rain, surface water	[74]
Nonylphenol	PF-FIA	--	[75]
Nonylphenol	ELISA	Water	[76]
2,4-dichlorophenoxy acetic acid	MIP-FIA*	--	[77]
	ELISA	Water	[78]

*MIP-FIA = molecularly imprinted fluorescent immunoassay

Hormones and pharmaceuticals belonging to the group of phenolics (see Table 3 for some examples) may be present in the environment and especially surface water and sewage treatment plant effluents as results of human and veterinary metabolism and drug use. An overview of the presence of such compounds was reported for the Dutch situation [79,80]. Worldwide pollution by human and veterinary hormones and pharmaceuticals and in particular estrogenic compounds have gained much interest. Various immunoassays have been developed or adopted from clinical assays for monitoring purposes based on observations regarding disturbed development and reproduction of wildlife, sex interchanges in e.g. molluscs, alligators and other animals, the generation of female treats in male fish etc. Examples of immunoassays include those for natural steroid hormones and metabolites (endogenous thyroid hormones are very unstable and will not be found in the environment) and pharmaceuticals used in medicine. Several studies were conducted to measure estrogens in wastewater, surface water, drinking water and sewage treatment plant effluents. Two different fluorescent immunoassays were described by Coille *et al.* [81] for measurement in spiked wastewater, showing a detection limit of 0.01 – 0.85 µg/L for estrone, estradiol and ethynylestradiol. A further interesting group comprises antibiotics belonging to the beta 2-agonist that are widely used in veterinary practice. These include, for example, salbutamol, terbutaline, pirbuterol, fenoterol, procaterol and formoterol. They are found in urine, blood, tissue and surface water. Due to strict regulations about maximum admitted levels in food products, various immunoassays have been developed for dectection, quantification and monitoring of these antibiotics. For example, an ELISA for fenoterol/ractopaine in calf urine was described by Haasnoot *et al.* [82], showing a detection limit of 0.2 ng/L. Monoclonal antibodies were used by Pou *et al.* [83] for the measurement of salbutamol/clenbuterol in a chemiluminescent EIA following immunoaffinity chromatography of tissue samples. Additional immunoassays for steroids and pharmaceuticals are summarized in Table 7.

**Figure 5:**
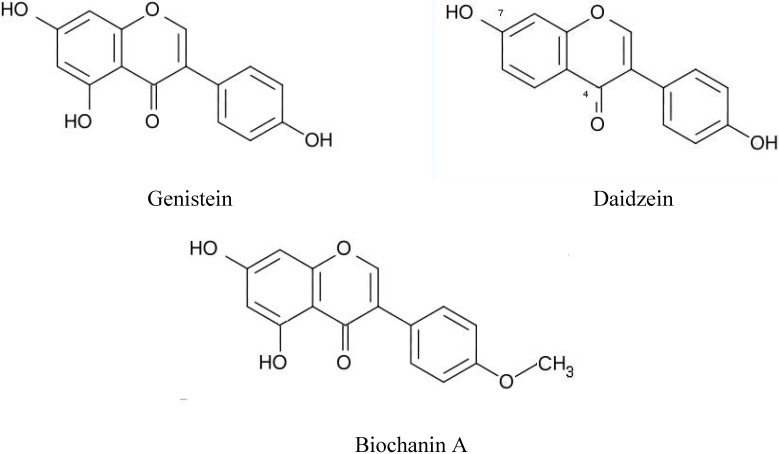
Chemical structure of three phytoestrogens. The positions 4 and 7 are given only in the figure for daidzain.

Phytochemicals as being beneficial to human health have attracted much interest and in particular those showing estrogenic activity. In this respect the above mentioned conventional analytical methods have been applied to determine levels of phytoestrogens in food and biological fluids. However, for large population studies HPLC does not show sufficient sensitivity, whereas GC/LC-MS is too laborious for screening of large numbers of samples. For this reason, immunoassays seem highly suitable and with regard to immunoassays for phytochemicals or phytoestrogens, the group of Adlercreutz and Lapčik has developed initially radioimmunoassays for daidzein, genistein, coumestrol, biochanin A measurement in blood and urine of different populations in relation to the intake of food (soy products) [91,92,93,94]. To be able to discriminate between these various isoflavonoids of interest that show chemical similarity (see Figure 5) the immunogens used for the production of antibodies should retain the moiety differing between the compounds exposed.

**Table 7:** Additional immunoassays for hormones and pharmaceuticals.

Target Compound	Format	Application	Reference
Estrone, estradiol	MIP-RIA		[84]
Estriol	RIA	serum	[85]
Dopamine	ELISA	serum	[86]
Cortisol	FIA	metabolism	[87]
Cortisol	RIA	blood	[88]
Paracetamol	FP-FIA	plasma	[89]
E2, EE2	ELISA	water	[90]

This means that this moiety should not be used for conjugation to carrier protein. For the respective radioimmunoassays the immunogens as well as the radioactive tracers were based on 4’-*O*-carboxymethyl or 7-*O*-carboxymethyl derivatives and ^125^I. Subsequently, radioimmunoassays were converted into non-radioactive, time-resolved fluoresencent immunoassays (TR-FIA) using europium (Eur) chelate as signalling tag to the same derivatives [95,96]. The results of the validation of these TR-FIAs are summarized in Table 8. Validation included the determination of the usual parameters to specify an antibody and/or immunoassay as well as comparison with reference methods (GC-MS).

**Table 8:** Parameters of non-radioactive immunoassays for daidzein (Dai), genistein (Gen), biochanin A (Bio).

	Dai	Gen	Bio
	*TR-FIA*	*TR-FIA*	* ELISA*
Immunogen	4’-CM-Dai^1^	4’-CM-Gen^1^	7’-CM-Bio^1^
Tracer	4’-CM-Eur	4’-CM-Eur	2^nd^ Ab-PO^2^
Detection limit	1.0 nM	1.7 nM	5.3 pg
Working range	1.0-216 nM	1.7-370 nM	5-100 pg
Precision			
Intra-assay	3.8-5.8	3.7-7.2	14.4 % C.V.
Inter-assay	7.9-9.2	7.6-9.8	11.9 % C.V.
Recovery			
Cross-reactivity (%)			
Enterolactone	-	-	<0.01
Enterodiol	-	-	<0.01
Matairesinol	-	-	-
Anhydrosecoisolaciresinol	-	-	-
Secoisolariciresinol	-	-	-
Daidzein	100	2.5	<0.01
Formononetin	206	44.4	0.3
Biochanin	3.5	500	100
Daizein	6.0	1.0	<0.01
Dihydrodaidzein	3.1	0.1	<0.01
Genistein	1.1	100	2.8
Dihydrogenistein	0	11.3	-
Genistin	0	7.6	<0.01
Equol	0	0.1	<0.01
O-Desmethylangolensin	0	0	-
Luteolin	0	0	-
Quercetin	0	0	-
Sissotrin	-	-	120
Prunetin	-	-	5.0
5-Methoxygenistein	-	-	<0.01

^1^ CM means the carboxymethyl derivative of the particular hapten compound ^2^ PO means peroxidase conjugated to the antibody (Ab)

Then, these assays were applied in studies into the relation between food intake or isoflavone administration and concentrations present in blood and/or urine of various dietary and population groups [97]. It was found that levels of the phytoestrogens enterolactone, genistein and daidzein were significantly higher in urine in vegetarian than in omnivorous healthy women with mean concentrations of 4.9 – 0.49 – 0.56 µg/L vs. 8.82 – 1.68 – 2.12 µg/L, respectively. Because isoflavonoids in foods are predominantly in glycoside form and isoflavonoids in urine mostly in glucuronide and sulfate form, sample pretreatment by hydrolysis and extraction was required before analysis. It was found that soy-based food intake resulted in higher concentration of the phytochemicals possessing estrogenic activity and metabolites thereof in body fluids, which may explain the lower incidence of hormone-related disorders and cancers in vegetarian or Asian populations in comparison with Western populations [98]. In another study of the same working group monoclonal antibodies against the same phytoestrogens daidzein, genistein and equol were produced and these are used for the determination in blood and urine in a Isoheart intervention study for the investigation of cardioprotective properties of isoflavones [99]. Similarly, ELISAs were developed for the measurement of daidzein and its biological most potent metabolite equol in plasma extracts [100]. Polyclonal antibodies were raised against immunogen-KLH conjugates. The ELISAs were validated by determining the detection limit (0.2 ng/well for daidzein and 0.07 ng/well for equol), cross-reactivities, precision and matrix effect.

Taxol, an anti-cancer compound isolated from the bark of *Taxus brevifolia* that may be effective against ovarian and breast carcinomas as well as carcinomas, was the target compound for the development of both an ELISA and a RIA for measurement in various plant extracts in a search for alternative sources [101]. 

As mentioned above, mycotoxins derived from some fungi that may be present on cereals (wheat, corn, peanut) and, consequently, in foodstuffs are highly toxic and have urged the development of specific and sensitive methods, including immunoassays. In food, the application of kits for mycotoxins yielded levels of 80 ng/g of deoxynivalenol, 30 ng/g of T-2 toxine and 80 ng/g of nivalenol [102]. Usleber *et al.* used an enzyme immunoassay for food samples and they found a concentration of 20 µg/L in wheat [103]. Beer appeared to be contaminated with 4.0 to 56.7 ng/mL of deoxynivalenol [104], whereas a series of red wines showed mean levels of 0.64 µg/L of ochratoxin A and 0.07 to 1.36 µg/L of resveratol [105]. Zeranol is a metabolite of zearalenon, a *Fusarium* toxin, and exhibits activity as anabolic agent. A time-resolved fluorescent immunoassay (TR-FIA) using a polyclonal antibody was applied by Tuomola *et al.* [106] for the screening of bovine urine. From cross-reactivities it appeared highly specific having a detection limit of 0.16 ng/mL. Other immunoassays developed for phytochemicals are summarized in Table 9.

**Table 9:** Additional immunoassays for phytochemicals.

Target Compound	Format	Application	Reference
Enterolactone	TR-FIA	plasma	[107]
O-desmethyl-angolensin	TR-FIA	plasma/urine	[108]
THC (cannabis)	ELISA	--	[109]

### Binding assays

In this context, binding assays comprise competitive assays using the hormone-binding transport proteins SHBG (Sex Hormone Binding Globulin), CBG (Corticosteroid Binding Globulin), TTR (Transthyretrin) and TBG (Thyroid Binding Globulin) in conjunction with a tracer. These transport proteins are present in blood and after release of newly synthesized steroid/thyroid hormones they bind them for more than 90 %. The role of these transport proteins is to protect the bound hormones against degradation and transport them to their respective target organs. In the last quarter of the last century, the Free Hormone Hypothesis was postulated by Ekins [110] and Mendel [111]. According to this hypothesis only the free fraction of steroid and thyroid hormones are biologically active and the equilibrium between protein-bound and free levels is a delicate one that defines the hormonal status of an organism. Any substance that disturbs this equilibrium by displacing bound hormones will have an influence on the hormonal status and may lead to hormone-related disorders and diseases, including cancer. In addition, an increase in the level of one or more of these transport proteins may also affect free concentration of the corresponding hormones. It has been known that e.g. elevated concentrations of estrogens during pregnancy or oral contraceptive use induce the synthesis of SHBG and CBG in the liver. A similar effect may be exerted by phytoestrogens and this may explain their beneficial effects on hormone-related disorders, menopause and cancers. Therefore, transport proteins have been used in investigations into endocrine disruptive effects of environmental pollutants and phytoestrogens.

The group of Hammond in Canada focussed on SHBG, demonstrating interactions between plasma SBG and natural, pharmaceutical and xenobiotic ligands, including synthetic estrogens, several phytochemicals and OH-PCB congeners [112]. Estrogenic activity was deducted from the ability of any compound to displace radiolabeled estradiol from SHBG. It appeared that from the phytochemicals tested none bound to SHBG with sufficiently high affinity for measurement. In our own patented method [113] we designed a competitive binding assay comprising anti-SHBG antibody as capturing component, SHBG as binding component and tritiated estradiol as tracer for the determination of the affinity of various pesticides for SHBG and a possible estrogenic effect. The relation between dietary fiber intake, urinary lignans and phytoestrogens in food with the blood level of SHBG and the reciprocal concentration free estradiol has been demonstrated in various female dietary and population groups by Adlercreutz *et al.* [114]. The association of phytoestrogens and several genes, including the SHBG gene, showed protection against prostate cancer risk, which has been reported by Low *et al.* [115]. 

CBG is the transport protein for cortisol or in rodents its counterpart corticosterone. Surprisingly, progesterone is also bound by CBG at the one binding site. Free cortisol has been demonstrated to be elevated in e.g. pregnancy, when progesterone increases dramatically up to delivery and displaces bound cortisol. In depression and stress situations both total cortisol and free cortisol in plasma are above normal with CBG being in the normal range. Until now CBG has not been used in studies investigating effects of natural, pharmaceutical or industrial to the same extent as SHBG or TTR/TBG, despite the fact that glucocorticoids play important roles in development, metabolism and mental health.

Thyroid mimetic environmental pollutants have been subject of the PhD study of Marchesini [116] because of possible effects on development, brain function and metabolism. By using the transport proteins TTR and TBG a competitive bioassays was developed and numerous compounds were analyzed on affinity for these proteins. It appeared that in particular hydroxylated PCB and dioxines showed a thyroid-like activity.

### Immuno Affinity Chromatography

The detection of compounds of interest in a complex matrix at sometimes very low levels poses a problem in many analytical methods. In addition to various pretreatment techniques such as selective liquid-liquid, solid-liquid or membrane extractions, antibodies may offer a means to solve this problem. Immunoaffinity chromatography is a technique wherein any antibody is covalently coupled to a solid phase such as silica, alumina, sepharose, agarose. Then this Ab-bound support material is packed into a column. Sample containing compounds to be analyzed is passed through the column whereby only those compounds recognized by the antibody are retained and others flow through. The bound compound(s) may then be eluted such that the antibodies may optionally retain binding activity and the immunoaffinity column be used more than once. The advantage of immunoaffinity chromatography (IAC) is the possibility to purify, isolated and concentrate particular compounds from complex samples, in particular for clean-up before analysis. There have published several reviews about IAC in general [117], basic principles [118], detection techniques [119], application in environmental monitoring [120] and as pretreatment of samples before GC-MS [121]. The technique has been used in both environmental and food analysis. Examples are IAC columns for multi-analyte isolation [122,123] or selective compounds such as phenylurea and triazine pesticides, steroid estrogens, and zearalenone [124,125]. A rapid field device based on IAC columns was developed by Goryacheva *et al.* [126,127]. Herein, the complete immune reaction plus colour development can be performed on the columns. Using this technique, ochratoxin A was detected in herbs and spices at cut off levels of 10 µg/kg. Further IAC columns and their application for the purification and concentration of mycotoxines from foodstuffs were described for aflatoxin M1 in milk [128], zearalenone in grains [129,130], pesticides and toxins in corn [131], or mycotoxins in cereals [132]. Application for the detection of drugs in tissues and biological fluids were already described by Katz and Siewiersky [133], whereas beta-agonists were subject of a report of Jeannet and Rohrbasser [134].

### Biosensors

In the last two decades biosensors have gained much interest at the field of environmental and food analysis as monitoring tools. Biosensors are devices that combine a biological component and a transducer for the transfer of a biological reaction signal. Because in biosensors the same components as above may be used as biological recognizing elements, biosensors may be considered as highly advanced measuring devices based on antibodies, enzymes, receptors and binding proteins or even DNA (aptamers) and whole cells. The biological element renders high selectivity and sensitivity in an biosensor assay. Depending on the recognition element the biological reaction leads to a change in current, conductivity, temperature, mass, wave length, colour, fluorescence or (chemi)luminescence. The mode of detection may therefore include electrochemical, thermal, optical or piezoelectric detection with signal processing. Many reviews about the various types of biosensors as well as surveys of commercially available optical biosensors have been published [135,136,137,138,139,140,141,142,143,144]. Herein the advantages, disadvantages, biocomponents, transducer types, coupling chemistry, target compounds, detection range and sample matrices are discussed. In the context of phenolics detection in environmental and food samples, some of these biosensors are particularly interesting, among which those based on enzymes, antibodies, receptors and binding proteins. One specific type of enzyme sensor is based on tyrosinase for the amperometric detection of polyphenols, e.g. in green tea, grape and olive oils with a detection range of 10 – 100 µmol/L [145]. A comparable tyrosinase-based biosensor was used for the determination of phenolic compounds such as catechol, *p*-cresol, phenol, *p*-chlorophenol and *p*-methylcatechol in water samples at very low detection limits [146]. A special biosensor with immobilized tyrosinase was developed for the enzymatic determination of BPA in aqueous solution [147]. In optical biosensors both direct and indirect detection formats have been developed. An interesting direct format includes the SPR (surface plasmon resonance) sensor based on the detection of changes in mass on the sensing surface. In this respect the Biacore system is nowadays a valuable tool in research and monitoring studies. For an overview of different optical biosensors, see Fan *et al.* [147]. For the environmental analysis of EDC showing thyroidal activity a SPR biosensor was developed based on TTR and TBG by Marchesini [116]. The most sensitive format appeared to be the one wherein a T4-derivative was covalently coupled to the sensing surface, whereupon sample together with TTR or TBG was passed through the sensor. The presence of compound to be tested inhibited binding of ligand to the sensing surface and the absence of change in mass. In this study a series of compounds suspected to have thyroidal activity have been tested and it appeared that in particular hydroxylated PCB and dioxines showed such activity. Similarly, an immune biosensor assays was developed for BPA by covalently coupling a BVA-spacer-conjugate to the sensing surface and passing sample and monoclonal or polyclonal antibody using a simplified, low cost SPR sensor [148]. An example of an indirect biosensor is the immune sensor developed in the RIANA project for the monitoring of pollutants in surface water. Herein antibodies are covalently coupled to the sensing surface, sample and fluorescent tracer are added for competitive reaction on the antibody layer and a fluorescent signal is measured at the end of the sensor device. In this design several pesticides and EDC have been measured on line in the river Rhine [149,150,151]. Detection limits for the target compounds atrazine, BPA, estron, isoproturone, alachlor, 2,4-D, simazine and PCP were mostly below 0.1 µg/L, which is the official European norm for pollutants in surface water intended for the production of drinking water. The detection limit achieved was 0.1 – 0.4 µg/L. Finally, a review about immune sensor based on on-line capture of target compounds and various detection systems for environmental pollutants was published by Gonzales *et al.* [152]. It will be apparent that sensors, and in particular immune sensors, are highly suitable for the real-time monitoring of specific compounds, even in complex matrices.

## Conclusions and future perspectives

From this review it will be clear that many (poly)phenolic compounds are present in the environment, food/feed and biological fluids. Concerns about health effects pertain in particular to the anthropogenic or industrial compounds because they are inherently toxic or endocrine disruptive. There has been put much effort in the development of analytical methods for such substances, in particular with regard to those compounds that may be present in surface water used for drinking water production due to various regulations. Pesticides, for example, should not exceed a norm level of 0.1 µg/L for single compounds and 0.5 µg/L for the sum of pesticides. Although conventional methods such as HPLC, GC, and MS are used predominantly for the quality control of water and soil, immunochemical methods have also been developed and used especially for large numbers of samples and monitoring purposes. Recently, attention has shifted from the detection and quantification of pollutants to the assessment of a biological effect, including an endocrine effect of samples, followed by the identification of causative compounds. From the numerous compounds tested and found active, but some stand out, such as alkylphenols, bisphenol A, hydroxylated PCBs and dioxine, because they are ubiquitous and they can affect several hormonal systems. Exposure and especially combination effects might explain the increased incidence of some modern health problems such as obesitas, diabetes, metabolic syndrome, depression, hormone-related cancers, etc.

Endogenous hormones and pharmaceuticals are known to have an effect on humans and animals. However, due to excretion or spills or the use of contaminated sludge such compounds may also be present in water, soil or food/feed; although mostly in low concentrations, ingestion of combinations with additive or even synergic actions should be of interest for control agencies and the public. With regard to analytical methods the same applies as for industrial compounds.

Phytochemical phenolics present a different class of compounds present in plants and trees. In contrast to the predominantly negative effects of the compounds listed above, phytochemicals may be beneficial, health-promoting and act as relieving agents for various diseases and even cancer. They have been used in medicinal compositions for centuries, but the exact nature of the active components has only been determined recently and for only part of the thousands of compounds. Vegetables and fruits are especially rich in beneficial phytochemicals and that emphasizes their intake for a healthy life. The relation between the intake of phytochemicals, concentrations in biological fluids and health-promoting effects involves food-population studies. However, until now these have been performed only sparsely. Conventional analytical methods are not suitable for such studies and immunoassays in combination with certain bioassays might be a good alternative. Unfortunately, the number of immunoassays for phytochemicals is limited and bioassays are mainly restricted to those for the assessment of estrogenicity.

Thus, there is a need for new bioassays and immunoassays, including immunosensors for real-time and sensitive detection and optionally immunoaffinity supports and sensors for purification, concentration and real-time detection of target compounds. Further investigations may provide new components for food, neutraceuticals or drug leads for pharmaceutical application.

## Supplementary Files

Supplementary File 1
