# Correction: Meulenberg, E. P. Phenolics: Occurrence and Immunochemical Detection in Environment and Food. *Molecules* 2009*, 14,* 439-473

**DOI:** 10.3390/molecules140803018

**Published:** 2009-08-13

**Authors:** Eline P. Meulenberg

**Affiliations:** ELTI Support VOF, Drieskensacker 12-10, 6546 MH Nijmegen, The Netherlands

In the original published version of this paper [1], there are two repeated refs. 134 in the reference list. We have now corrected the second ref. 134 to ref. 135, and the remainder should to be renumbered accordingly.
