# Supplementary material for: Phenolics: Occurrence and Immunochemical Detection in Environment and Food
Source: Molecules. 2009 Jan 19;14(1):439–73. doi: 10.3390/molecules14010439 (PMC6253769; doi:10.3390/molecules14010439)
Supplement: Supplementary File 1 [file molecules-14-00439-s001.pdf]

Molecules **2009**,*14*, 3018; doi:10.3390/molecules140803018

OPEN ACCESS

molecules

ISSN 1420-3049

www.mdpi.com/journal/molecules

Correction

Correction: Meulenberg, E. P. Phenolics: Occurrence and Immunochemical Detection in Environment and Food. *Molecules* 2009, *14*, 439-473

Eline P. Meulenberg *

ELTI Support VOF, Drieskensacker 12-10, 6546 MH Nijmegen, The Netherlands

* Author to whom correspondence should be addressed; E-mail: E.Meulenberg@eltisupport.nl

Received: 3 August 2009 / Published: 13 August 2009

In the original published version of this paper [1], there are two repeated refs. 134 in the reference list. We have now corrected the second ref. 134 to ref. 135, and the remainder should to be renumbered accordingly.

Reference

1. Meulenberg, E. P. Phenolics: Occurrence and Immunochemical Detection in Environment and Food. *Molecules* **2009**, *14*, 439-473.

© 2009 by the authors; licensee Molecular Diversity Preservation International, Basel, Switzerland. This article is an open-access article distributed under the terms and conditions of the Creative Commons Attribution license (http://creativecommons.org/licenses/by/3.0/).
